# Dermoscopic Features of Two Cases of Angiolymphoid Hyperplasia with Eosinophilia and Review of the Literature

**DOI:** 10.5826/dpc.1102a03

**Published:** 2021-03-08

**Authors:** Bengu Nisa Akay, Mehmet Fatih Atak, Banu Farabi

**Affiliations:** 1 Dermatology Department, Ankara University School of Medicine, Turkey; 2 Dermatology Department, Rutgers University, Robert Wood Johnson Medical Center, New Jersey, USA

**Keywords:** angiolymphoid hyperplasia with eosinophilia, red clods, dermoscopy

## Introduction

Angiolymphoid hyperplasia with eosinophilia (ALHE) is a rare benign vascular tumor that typically presents as papules or nodules that are pink or dull red in color and are located predominantly in the head and neck region. Mural damage or rupture of large intralesional vessels suggest that trauma or arteriovenous shunting may play a etiological role, and a history of trauma can be elicited in some patients. The lesions may be asymptomatic or may be painful, pruritic, bleeding or pulsatile. Some patients have regional lymph node enlargement and peripheral eosinophilia.

Clinical diagnosis of this entity can be challenging especially in atypical presentations and dermoscopy might be helpful when considring ALHE as a differential diagnosis. To date, dermoscopic features of ALHE have been described in 3 cases [1,2]. Herein, we present 2 cases of ALHE with their dermoscopic features.

## Case Presentations

**Figure 1:**
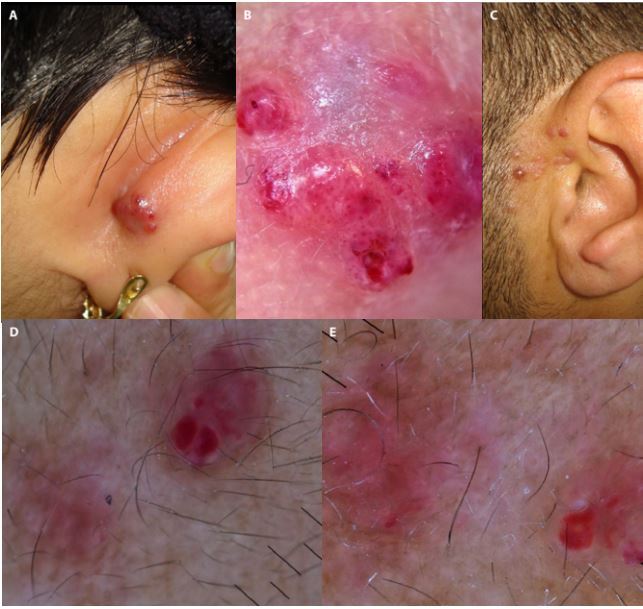
Clinical and dermoscopic pictures of angiolymphoid hyperplasia with eosinophilia. (A) A 46-year-old woman with a multinodular reddish lesion on the right posterior auricular area. (B) Dermoscopic image shows red clods with dotted vessels, subtle white lines and pink structureless areas. (C) A 34-year-old man with multiple pink-colored papules in the left preauricular area. (D, E) Dermoscopic images show a focal area of red clods and subtle white lines and a peripheral brown structureless area.

The reported characteristics and dermoscopic findings of our patients are presented in Table 1. Clinical and dermoscopic images are shown in Figure 1. Both of our patients’ diagnoses have been confirmed by histopathological examination.

## Discussion

ALHE is a rare benign vasoproliferative disorder of unknown origin. Various hypotheses have been described including prior trauma, reactive hyperplasia, benign neoplasia, hyperestrogenemia, infectious agents, and atopy.

Although ALHE is a benign entity, it can be confused with many conditions including sarcoidosis, lymphocytoma cutis, Kaposi sarcoma, angiosarcoma and metastatic tumors clinically. Therefore, biopsy is often required for diagnosis.

Dermoscopic features of ALHE are described in 2 case reports [1,2]. Rodriquez et al. observed a polymorphous vascular pattern consisting of dotted and linear vessels on a pale reddish to pinkish background [1]. Santosa et al. described a clinical mimicker of keratoacanthoma, showing a central keratin mass and ulceration surrounded by dotted, globular and linear irregular vessels on dermoscopy [2]. In our first case case we observed large red clods with dotted vessels, subtle white lines and pink structureless areas. In the previous reports of ALHE red clods were not observed. Our second case also presented with a focal area of red clods and subtle white lines and serpentine-looped vessels arranged on a pale pink-brown structureless area.

## Conclusions

Pink lesions represent a challenge for dermatologists and dermoscopy has proven to be a valuable tool in improving the diagnosis of cutaneous neoplasms in comparison with examination with the naked eye. Although pink structureless areas, polymorphic vessels and white lines observed in our 2 cases can be observed in other pink nodular lesions, namely, amelanotic melanoma, Spitz nevus, and dermatofibroma, the presence of red clods is helpful in differentiating a vascular lesion from the aforementioned entities. Other vascular tumors such as angiosarcoma and Kaposi sarcoma are the major differential diagnoses for ALHE. In Kaposi sarcoma the presence of large red clods is not expected as observed in our cases. Also, red clods are not reported in angiosarcoma.

In conclusion, considering the typical location and clinical findings, ALHE should be kept in mind as a differential diagnosis in the presence of dermoscopic red clods.

## Figures and Tables

**Table 1:** Comparison of Case 1 and Case 2 with Current Cases Reported in the Literature

	Age (Years)	Sex	Location	Duration	Clinical Features	Preceding Trauma	Dermatoscopy	CBC and USG Findings
Case 1	46	Female	Right posterior auricular area	3 months	Asymptomatic, multinodular, reddish lesion	No history of trauma	Large sized red clods with randomly arranged short white lines and regular dotted vessels over each clod	Normal
Case 2	35	Male	Left preauricular area	8 months	Pruritic, multiple pink-colored papules	No history of trauma	A focal area composed of red clods, pink-brown structureless areas with serpentine and looped vessels, subtle white lines	Normal
Rodriguez-Lomba et al. Case 1	45	Male	Inner aspect of the left thigh	6 months	Asymptomatic, enlarging, pink-colored nodule	Not mentioned	Polymorphous vascular pattern composed of dotted, corkscrew, and irregular-linear vessels arranged radially over a diffuse pale reddish background	Not mentioned
Rodriguez-Lomba et al. Case 2	17	Female	Front of the neck	Recent onset	Asymptomatic, pink-colored multinodular proliferation	Not mentioned	Dotted and irregular vessels regularly distributed within each nodule over a diffuse light pink background	Not mentioned
Santosa et al.	55	Male	Nose	7 months	Bleeding, 5-mm diameter lesion with solitary ulcer	Electrocautery performed to the lesion considered keratoacanthoma, 1 year previously	Multiple dotted vessels and central ulceration with adjacent white area	Not mentioned

CBC = complete blood count; USG = ultrasonography.
